# Pregnancy- and age-associated variation in serum dehydroepiandrosterone concentrations in black and white rhinoceroses

**DOI:** 10.1093/conphys/coag007

**Published:** 2026-02-12

**Authors:** Drew M Arbogast, Lara C Metrione, Marieke K Jones, Elizabeth M Donelan, Terri L Roth, Elizabeth W Freeman, Louisa A Rispoli

**Affiliations:** Department of Environmental Science and Policy, George Mason University, 4400 University Drive, Fairfax, VA 22030, USA; South-East Zoo Alliance for Reproduction and Conservation, 581705 White Oak Road, Yulee, FL 32097, USA; Department of Biology, George Mason University, 4400 University Drive, Fairfax, VA 22030, USA; Center for Conservation and Research of Endangered Wildlife, Cincinnati Zoo and Botanical Garden, 3400 Vine Street, Cincinnati, OH 45220, USA; Center for Conservation and Research of Endangered Wildlife, Cincinnati Zoo and Botanical Garden, 3400 Vine Street, Cincinnati, OH 45220, USA; School of Integrative Studies, George Mason University, 4400 University Drive, Fairfax, VA 22030, USA; Center for Conservation and Research of Endangered Wildlife, Cincinnati Zoo and Botanical Garden, 3400 Vine Street, Cincinnati, OH 45220, USA

**Keywords:** Age, biomarker, enzyme immunoassay, gestation, parturition, reproduction

## Abstract

Dehydroepiandrosterone (DHEA) is an important hormone precursor for androgen and oestrogen sex steroids, yet it is understudied in wildlife and has not been measured in rhinoceroses. The objective of this study was to examine serum DHEA concentrations in *ex situ* black (*Diceros bicornis*; *n* = 22 male, 18 female) and white (*Ceratotherium simum*; *n* = 25 male, 46 female) rhinoceroses. A commercially available DHEA immunoassay was validated for use with rhino serum, and monthly samples (*n* = 1029) were analysed. Analytical validation included demonstrating parallel displacement curves between serially diluted standards and pooled extracts, as well as 91% extraction efficiency in a spike and recovery test. Differences in DHEA concentrations relative to species, age, sex and pregnancy status were analysed using linear mixed models. Serum DHEA concentrations were higher (*P* < 0.001) in black (194 ± 14.2 pg/ml) versus white (123 ± 8.0 pg/ml) rhinoceroses and demonstrated a non-linear relationship with age in both species, with concentrations peaking around 15 years of age before declining thereafter. No sex differences between males and non-pregnant females were detected in either rhinoceros species. White rhinoceros DHEA concentrations were higher (*P* < 0.001) in pregnant (309 ± 31.9 pg/ml, *n* = 15) compared to non-pregnant (120 ± 10.4 pg/ml, *n* = 41) females; pregnant black rhinoceroses similarly produced elevated DHEA concentrations during pregnancy (1092 ± 90.3 pg/ml; *n* = 2) compared to non-pregnant (229 ± 8.1 pg/ml; *n* = 17) females. DHEA concentrations also increased throughout gestation particularly during mid- to late gestation in both species. These findings provide new insight into rhinoceros endocrinology and suggest potential utility of DHEA for monitoring pregnancy status.

## Abbreviations

DHEA, dehydroepiandrosterone
DHEA-S, dehydroepiandrosterone sulphate
EIA, enzyme immunoassay
DE, diethyl ether
EA, ethyl acetate
ACN, acetonitrile
NE, non-extracted
CV, coefficient of variation
AIC_c_, corrected Akaike Information Criterion

## Introduction

The critically endangered black rhinoceros (*Diceros bicornis*) and the near-threatened white rhinoceros (*Ceratotherium simum*) are at risk of disappearing from the wild due to poaching for their horns, habitat loss and degradation and slow reproductive rates (Ferreira *et al.*, 2022; Schwarzenberger and Hermes, 2023). Conservation efforts in zoos and wildlife reserves are crucial for saving these species, and studying specific physiological biomarkers in these controlled environments can reveal important insights related to rhinoceros health and well-being (von Houwald *et al.*, 2025). For example, dehydroepiandrosterone (DHEA) is a biomarker of ageing in some taxa, as concentrations decline with age in horses (*Equus caballus*, Satué *et al.*, 2024), dogs (*Canis lupus familiaris*, Marinelli *et al.*, 2007; Mongillo *et al.*, 2014) and many primate species (*Papio hamadryas*, *Macaca mulatta*, Goncharova and Lapin, 2002; *Macaca nemestrina*, *Papio cynocephalus*, Muehlenbein *et al.*, 2003; *Macaca fuscata*, Takeshita *et al.*, 2013). Additionally, elevated serum DHEA is linked to musth behaviour and physiology in Asian elephants (*Elephas maximus*, Yon *et al.*, 2007), and faecal DHEA metabolite concentrations obtained from healthy cetaceans in human care (*Tursiops truncatus*, *Tursiops aduncus*, *Lagenorhynchus obliquidens*, *Delphinapterus leucas*) serve as valuable reference intervals for veterinary professionals assessing cetacean health (Miller *et al.*, 2021).

DHEA and its sulphated derivative DHEA sulphate (DHEA-S) are two steroid hormones produced by the adrenal glands and involved in the stress response system (Kamin and Kertes, 2017). Often referred to as a glucocorticoid antagonist, DHEA works to counteract the immunologic and neurotoxic effects of glucocorticoids (Kimonides *et al.*, 1999; Maninger *et al.*, 2009; Kamin and Kertes, 2017). In humans (*Homo sapiens*), both DHEA and DHEA-S play important regulatory roles for improving cellular immunity, endothelial function, memory and cognitive function, mental health, inflammatory response, neurogenesis and neuronal protection (Maninger *et al.*, 2009; Traish *et al.*, 2011). These regulatory effects also have been demonstrated in laboratory and domestic animal models, including a role in regulating vascular and metabolic health (dogs, Kurzman *et al.*, 1998; pigs, *Sus scrofa domesticus*, Molinari *et al.*, 2004), inhibiting pro-inflammatory cytokine production (mice, *Mus musculus*, Alves *et al.*, 2016) and reducing oxidative damage (dogs, Shen *et al.*, 2001; mice, Cao *et al.*, 2020).

Although primarily produced by the adrenal glands in mammals, both DHEA and its sulphated form are also synthesized by the gonads, placenta and brain (Gabai *et al.*, 2020). DHEA and DHEA-S are integral precursor hormones for androgen and oestrogen sex steroids, including androstenedione, testosterone and estradiol (Leowattana, 2004; Rainey *et al.*, 2004; Makieva *et al.*, 2014; Gabai *et al.*, 2020). Endogenous DHEA measurement may have utility for detecting and monitoring animal pregnancy (Hart *et al.*, 2023). Concentrations of faecal DHEA-S were elevated during late pregnancy in Japanese macaques (*M. fuscata*, Takeshita *et al.*, 2016), orangutans (*Pongo pygmaeus*, Takeshita *et al.*, 2019), howler monkeys (*Alouatta caraya*, da Silva *et al.*, 2021) and siamangs (*Symphalangus syndactylus*, Takeshita, 2022). In horses, serum DHEA rises early in pregnancy, peaks during mid-gestation and then declines progressively until parturition (Legacki *et al.*, 2016; Satué *et al.*, 2019). The elevated DHEA concentrations observed during mid-pregnancy are likely of foetal rather than maternal origin and are a presumed important source of oestrogens needed for gestation maintenance and parturition (Legacki *et al.*, 2017; Conley and Ball, 2019). Serum DHEA-S may also rise in pregnant mares, although concentrations appear much lower than those of DHEA and drop to undetectable concentrations by mid-pregnancy (Legacki *et al.*, 2019). Indeed, there is a general lack of information available on the function and origin of DHEA-S compared to DHEA, particularly in ungulates (Gabai *et al.*, 2020). This difference may be related to challenges in measuring circulating concentrations of DHEA and DHEA-S, which are markedly lower in non-primate mammals compared to primates (Rege *et al.*, 2019). Nonetheless, incorporating DHEA and DHEA-S measurements into existing monitoring practices could improve pregnancy diagnosis capabilities and gestation monitoring of animals in managed care.

Previous studies examined downstream salivary androgen and faecal androgen metabolite concentrations in greater one-horned rhinoceroses (*Rhinoceros unicornis*; Schwarzenberger *et al.*, 2000; Gómez *et al.*, 2004); however, circulating DHEA remains understudied in wildlife species (Whitham *et al.*, 2020), and its role in rhinoceros reproductive physiology is largely unexplored. The objectives of this study were to (i) validate an enzyme immunoassay (EIA) for measuring serum DHEA in black and white rhinoceroses (henceforth referred to as rhinos) and (ii) examine individual variation in serum DHEA concentrations based on species, sex, age and pregnancy status. Given the close evolutionary relatedness between horses and rhinos, we hypothesized that rhino DHEA concentrations would decline with age and increase during pregnancy. This investigation was a critical first step towards understanding the biological functions of DHEA in rhinos and exploring its potential as a biomarker of animal health and well-being.

## Materials and Methods

### Chemicals and reagents

Unless otherwise noted, chemicals were obtained from Thermo Fisher Scientific (Waltham, MA) or MilliporeSigma (St. Louis, MO). The commercially available EIA kit for DHEA was purchased from Enzo Life Sciences (Farmingdale, NY).

### Study animals

The study population consisted of 40 black (*n* = 22 male, 18 female) and 71 white (*n* = 25 male, 46 female) rhinos housed across 37 North American facilities (Table S1). The date of birth for each rhino was obtained from population studbooks (e.g. Ferrie, 2020; Capiro, 2021) and contacts at participating facilities. Ages ranged from 3 to 33 years old for black rhinos and 3 to 55 years old for white rhinos. Daily management and husbandry practices varied by facility but followed the general guidelines described in the Rhinoceros Husbandry Manual (Metrione and Eyres, 2014). Institutional Animal Care and Use Committee approval for this study was received from the Center for Conservation and Research of Endangered Wildlife of the Cincinnati Zoo and Botanical Garden (#22-173) and George Mason University (1833567-1).

### Sample collection

Serum samples (*n* = 14) for assay validations were collected opportunistically during routine procedures from black (*n* = 2 male, 3 female) and white (*n* = 3 male, 4 female) rhinos. Subsequently, monthly serum samples (*n* = 1029) were collected over a 1-year period (between April 2022 and April 2024) by each facility’s respective veterinary and animal care teams. Study rhinos were trained for voluntary participation in blood collection. A standardized collection protocol was provided to participating institutions: whole blood was collected into additive-free serum tubes without a gel separator (Covetrus, Dublin, OH) and then allowed to clot for 30–60 minutes at room temperature or 2–4 hours under refrigeration. After centrifugation to remove blood cells, the serum was stored at −20°C (*n* = 3–12 monthly samples per rhino; Table S1) until overnight shipment on dry ice to the laboratory for analysis.

### Sample extraction, immunoassay and analytical validation

Three extraction methods commonly used for steroid hormone analyses were compared: (i) Diethyl ether (DE; 2.5 ml) was added to 500 μl of pooled serum in a 13 × 100-mm glass tube. After vortexing for 5 minutes, the contents were allowed to separate into layers and then placed into a dry ice–methanol bath. The non-frozen supernatant containing the hormone was poured off into a 12 × 75-mm glass tube and evaporated under forced air. A second extraction was then performed as described above (i.e. 2.5 ml of DE added) on the same serum aliquot, and the resulting supernatant was added to the same 12 × 75 mm glass tube and likewise evaporated. The final dried extract was stored at −20°C until reconstituted in 500-μl assay kit buffer just prior to assessment. (ii) Ethyl acetate (EA; 2.5 ml) was added to 500 μl of pooled serum in a 13 × 100-mm glass tube, and the same extraction steps as described for DE were followed. (iii) High-performance liquid chromatography-grade acetonitrile (ACN; 900 μl) was added to 600 μl of pooled serum in a 1.7-ml microcentrifuge tube. The sample was vortexed briefly, incubated for 10 minutes at ambient temperature, vortexed again and then centrifuged (5 min, 17 000 × *g*). A portion of the supernatant (1.2 ml) was transferred to a 12 × 75-mm glass tube and evaporated under a vacuum. The dried extract was stored at −20°C until reconstituted and vortexed in 240-μl assay kit buffer just prior to assessment.

DHEA concentrations were measured according to the instructions provided by the assay kit manufacturer with an overnight incubation to maximize sensitivity. As specified in the kit protocol, the antibody exhibits 100% cross-reactivity with DHEA and 30% with DHEA-S, and assay sensitivity is 2.90 pg/ml. For each of the three extraction types and for non-extracted (NE) serum, dose–response curves were compared for parallel slopes between pools of serially diluted samples and serially diluted DHEA standard. The slopes were plotted as optical density (logit-scale) versus relative dose (log-scale) and analysed using a comparison of fits *F*-test; the linearity of each set of serial sample dilutions was assessed using linear regression to ensure consistent measurement of hormone across the assay’s range (GraphPad Prism version 10.4.0; GraphPad Software, Boston, MA). In addition, spiked (+2500 pg/ml DHEA) samples were extracted and assayed alongside non-spiked samples to determine how much DHEA was recovered using each of the three extraction types. Recovery was calculated as [(spiked concentration − non-spiked concentration) ÷ spiked concentration] × 100. Recoveries between 80% and 120% were considered acceptable.

As a final validation step, two alternative in-house assay buffers were tested to determine compatibility with the DHEA assay and to enable the use of reconstituted extracts in a separate hormone assay (data not shown). The in-house assay buffer (0.1% Tween-20, 13.7 mM Trizma, 86.4 mM Tris-hydrochloride, 150 mM sodium chloride, 0.09% ProClin 150, 0.1% Bovine serum albumin, pH 7.5 ± 0.05) was evaluated with and without 10 mM ethylenediaminetetraacetic acid (EDTA; modified in-house buffer) and compared against the kit-provided buffer. To assess effects on enzyme conjugate activity, each buffer was tested using the Total Activity control step from the DHEA assay protocol. Briefly, 5 μl of DHEA–HRP conjugate, diluted 10-fold in one of the buffers, was added to a microplate well followed by the addition of 200 μl of substrate solution. The plate was incubated for 3 hours at 37°C without shaking before 50 μl of stop solution was added. Optical densities were then compared across the three buffer types to evaluate signal development.

Following extraction validation, monthly serum samples were extracted using the DE method described above but adjusted to a 1:10 ratio (i.e. 3 ml of DE into 300 μl of serum). Extracts were reconstituted with 300 μl of in-house assay buffer without EDTA and assayed neat. To monitor assay performance, high and low control pools were prepared using the DHEA standard from the kit, targeting final concentrations of 3000 and 300 pg/ml, respectively. Aliquots of each control were stored at −20°C and included on every assay plate (*n* = 42 plates). Samples, standards and controls were run in duplicate. The intra-assay coefficient of variation (CV) was 6.0% ± 0.13%. Inter-assay CVs for the high and low controls were 6.7% and 12.0%, respectively.

### Statistical analyses

Rhino age on the day each sample was collected was calculated in relation to the individual’s date of birth. Female pregnancy status was determined retrospectively based on confirmed dates of parturition (*n* = 2 black rhinos, 15 white rhinos) and the average length of gestation for each species (461 days for black rhinos, 504 days for white rhinos; Schwarzenberger and Hermes, 2023). Samples from female rhinos were categorized as non-pregnant or pregnant. One white rhino that miscarried mid-pregnancy was excluded from statistical analyses. However, the breeding date recorded by the facility was used as the estimated date of conception, and her hormone profile was examined qualitatively to assess whether circulating DHEA patterns differed from those of females at similar stages of pregnancy.

Variations in serum DHEA concentrations by species, sex, age and pregnancy status were analysed using R version 4.4.2 (R Core Team, 2024) and the following packages: ‘lme4’ (Bates *et al.*, 2015), ‘lmerTest’ (Kuznetsova *et al.*, 2017), ‘ggeffects’ (Lüdecke, 2018), ‘car’ (Fox and Weisberg, 2019), ‘emmeans’ (Lenth, 2024), ‘sjPlot’ (Lüdecke, 2024), ‘performance’ (Lüdecke *et al.*, 2021). DHEA concentrations were log-transformed for all models and statistical tests to meet the assumption of residual homoscedasticity. Linear mixed models included rhino and facility identification as random effects to account for repeated, unbalanced measures. For all samples from males and non-pregnant female rhinos, an exploratory model was examined with species, age and sex as fixed effects to assess whether a species difference was present. Subsequent models investigated black and white rhinos separately and used corrected Akaike Information Criterion (AIC_c_; Burnham *et al.*, 2011) to compare model fits. Candidate models included sex and age individually, the interaction between sex and age and non-linear relationships between DHEA and age (i.e. natural splines with one or two knots, based on preliminary data exploration). Predicted DHEA concentrations were generated based on the best fit (i.e. lowest AIC_c_ within two units) and most parsimonious model for each species. Variance inflation factors (VIF) were calculated to assess the multicollinearity of predictor variables in each model. All VIF scores were less than 2, indicating multicollinearity was not present (Fox and Weisberg, 2019).

Additional linear mixed models were used to examine the effects of pregnancy status (i.e. non-pregnant or pregnant) and gestation (i.e. the number of days relative to parturition) on female DHEA concentrations, respectively. Black rhinos were excluded from these two models due to a limited sample size (*n* = 14 samples from 2 pregnant rhinos). Significant associations for all models were identified using a restricted maximum likelihood approach and type I analysis of variance *F*-tests with Satterthwaite approximation. Model residuals and goodness of fit were evaluated using diagnostic plots in R (Lüdecke, 2024). Goodness of fit was calculated and reported as the marginal coefficient of determination (*R^2^*_m_) such that values closer to one indicated stronger associations and higher explained variation of fixed effects, whereas values closer to zero indicated weaker associations and lower explained variation. Similarly, the conditional coefficient of determination (*R^2^*_c_) was used to quantify the amount of variation explained by both the fixed and random effects (i.e. individual- and facility-level variation). Log-transformed DHEA concentrations were back-transformed for reporting and visualizations. Results were considered significant at *P* < 0.05 and reported as estimated marginal means (i.e. the predicted mean after accounting for individual- and facility-level variation and holding other fixed effects constant) ± standard error of the mean, unless stated otherwise. Finally, DHEA concentrations from non-pregnant white rhino females of reproductive age were summarized to define a 95th percentile concentration threshold. This empirically derived concentration was then mapped onto the model-predicted gestational DHEA trajectory to estimate when predicted DHEA concentrations in pregnant females would exceed values characteristic of non-pregnant physiology.

## Results

**Figure 1:**
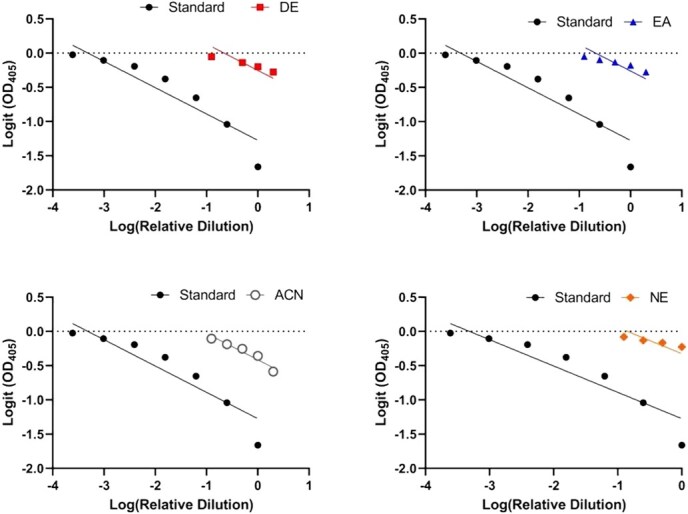
Parallel displacement curves (*F*_4,15_ = 1.73, *P* = 0.20) between serially diluted standard and pooled rhino serum samples (*n* = 5 black rhinos and 7 white rhinos) extracted via DE, EA, ACN and NE.

The assay detected DHEA in rhino serum across all tested sample extraction methods, including DE, EA, ACN and NE approaches. Each extraction method yielded parallel displacement curves between serially diluted standard and extracted sample pools (*F*_4,15_ = 1.73, *P* = 0.20; Fig. 1), as well as linearity across dilutions (*P* ≤ 0.01, *R^2^* > 0.91). Recovery for the spiked DHEA samples was 91% for DE, 78% for EA, 134% for ACN and 31% NE. Based on the combined results of the parallel and recovery tests, the DE extraction method was selected for use in the analysis of all study samples. In comparing buffer types, the optical densities of the Total Activity wells for the DHEA kit assay buffer, the in-house assay buffer and the modified in-house assay buffer without EDTA were 2.125, 0.647 and 2.311, respectively. Given that the modified assay buffer yielded results comparable to the kit-provided buffer, it was selected for resuspension of all extracted study samples.

**Figure 2:**
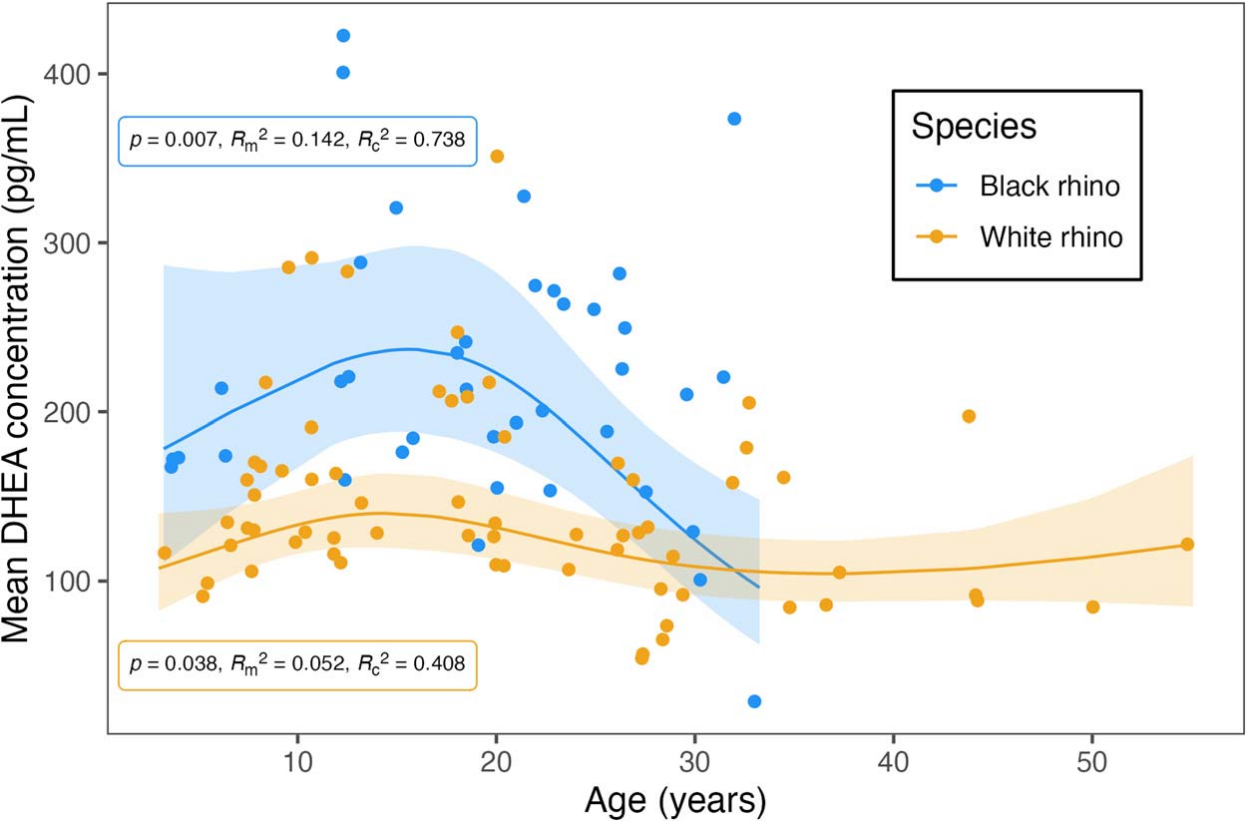
Associations between age and serum DHEA concentrations in male and non-pregnant female rhinos. The solid lines and shading indicate predicted DHEA concentrations and 95% confidence intervals, respectively, based on monthly sampling. Circles indicate the mean DHEA concentration per individual (*n* = 39 black rhinos, 67 white rhinos).

Considering only samples from males and non-pregnant females, DHEA concentrations were higher in black rhinos (194 ± 14.2 pg/ml, *n* = 356 samples from 39 rhinos) than white rhinos (123 ± 8.0 pg/ml, *n* = 549 samples from 67 rhinos; *F*_1,72.8_ = 28.751, *P* < 0.001). Sex was excluded from the best black rhino model but retained in the best white rhino model based on AIC_c_ values despite non-significance in both species (black rhino: *P* = 0.692; white rhino: *P* = 0.100). Interaction terms were similarly excluded based on AIC_c_. DHEA concentrations increased with age until about 15 years of age before declining thereafter in both black rhinos (*F*_2,34.2_ = 5.698, *P* = 0.007) and white rhinos (*F*_3,46.7_ = 3.051, *P* = 0.038; Fig. 2). Nonetheless, variation in DHEA concentrations was poorly explained by age (black rhino: *R^2^*_m_ = 0.142; white rhino: *R^2^*_m_ = 0.052) but improved when accounting for substantial individual- and facility-level variation (black rhino: *R^2^*_c_ = 0.738; white rhino: *R^2^*_c_ = 0.408; Fig. 2; Fig. S1).

**Figure 3:**
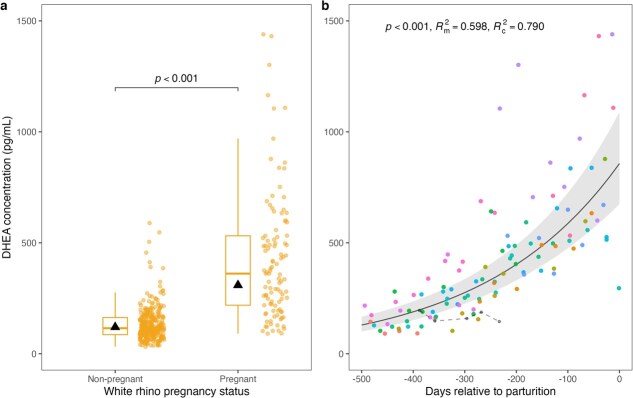
Associations between pregnancy and monthly serum DHEA concentrations in female white rhinos. (a) DHEA concentration versus pregnancy status in white rhino females (*n* = 45). Boxes and whiskers indicate the median, interquartile range, and values within 1.5 times the interquartile range. Circles indicate raw data points, whereas triangles indicate back-transformed estimated marginal means. (b) DHEA concentrations throughout gestation in pregnant white rhinos. Circles indicate raw data points with each colour representing an individual rhino profile. The solid line and shading indicate predicted DHEA concentrations and 95% confidence intervals, respectively, based on monthly sampling during successful pregnancies (*n* = 15). The dashed line represents samples from an additional white rhino that miscarried mid-pregnancy.

White rhino DHEA concentrations were higher in pregnant (309 ± 31.9 pg/ml, *n* = 105 samples from 15 rhinos) than non-pregnant (120 ± 10.4 pg/ml, *n* = 310 samples from 41 rhinos; *F*_1,192.5_ = 176.7, *P* < 0.001; Fig. 3a) females. Two pregnant black rhinos showed similar DHEA elevation (mean = 1092 ± 90.3 pg/ml, *n* = 14 samples from 2 rhinos) compared to non-pregnant (mean = 229 ± 8.1 pg/ml, *n* = 168 samples from 17 rhinos) females. White rhino DHEA concentrations also increased throughout gestation (*F*_1,83.2_ = 152.3, *P* < 0.001; Fig. 3b), and the variation was well explained by the model (*R^2^*_m_ = 0.598, *R^2^*_c_ = 0.790). The 95th percentile DHEA concentration for non-pregnant female white rhinos was 254.6 pg/ml. Model predictions indicated that gestational DHEA concentrations in pregnant females exceeded this value at ~321 days prior to parturition (95% confidence interval, −378 to −269 days), corresponding to roughly 183 days post-conception assuming a 504-day gestation period. In the white rhino that miscarried mid-pregnancy, DHEA concentrations remained relatively low (mean = 167 ± 10.3 pg/ml, *n* = 5 samples) across days 116 to 271 post-estimated conception (Fig. 3b). In addition, three white rhinos for which samples were collected within 30 days of parturition showed decreases (range = 29–44% decrease from previous monthly sample) in DHEA just prior to parturition.

## Discussion

This study provides the first evaluation of circulating DHEA concentrations in rhinos, revealing both age-related trends and pregnancy-associated elevations consistent with expectations based on equid physiology (Legacki *et al.*, 2016; Satué *et al.*, 2019, 2024). Sex did not emerge as a major explanatory factor among male and non-pregnant female rhinos despite DHEA concentrations in other species commonly being higher in males than females (dogs, Mongillo *et al.*, 2014; pigs, Bergamin *et al.*, 2019; humans, Turcu *et al.*, 2020). Although the species-specific mechanisms and functions of DHEA production are understudied in rhinos, and in wildlife generally, our results contribute to a growing body of work uncovering the potential of DHEA as a physiological biomarker for assessing animal health and reproduction.

DHEA concentrations were consistently higher in black rhinos than in white rhinos, suggesting possible species-level differences in adrenal steroid production, adrenal steroid metabolism or other aspects of rhino stress physiology. The higher circulating concentrations in black rhinos could be related to elevated glucocorticoid concentrations, which others have reported are higher in black than white rhinos (Brown *et al.*, 2001). As hypothesized, DHEA concentrations also varied by age in non-pregnant individuals of both rhino species. The median life expectancy of rhinos in North America is 20.2 years for black rhinos (observed maximum longevity = 52 years; Smith *et al.*, 2025) and 36.1 years for white rhinos (observed maximum longevity = 56 years; Eyres *et al.*, 2025). The increased DHEA concentrations during early adulthood until ~15 years of age may correspond to peak breeding age in rhinos (estimated 8–17 years old for black rhinos, Smith *et al.*, 2025; estimated 10–18 years old for white rhinos, Eyres *et al.*, 2025). In humans and great apes, adrenal androgen production similarly increases between puberty and mid-adulthood before declining with age (Havelock *et al.*, 2004; Campbell, 2006). The general trend for DHEA to decline in older rhinos also parallels patterns reported for mares (Satué *et al.*, 2024), dogs (bitches, Marinelli *et al.*, 2007; males and females, Mongillo *et al.*, 2014) and additional primate species (hamadryas baboons, *P. hamadryas*, rhesus macaques, *M. mulatta*, Goncharova and Lapin, 2002; rhesus macaques, pig-tailed macaques, *M. nemestrina*, and yellow baboons, *P. cynocephalus*, Muehlenbein *et al.*, 2003; Japanese macaques, Takeshita *et al.*, 2013). The white rhino model predicted a slight increase in DHEA concentrations after 40 years of age. However, this pattern appears to be driven by samples from a single individual, as samples from similarly aged white rhinos suggest DHEA remains relatively low after 30 years of age. Our sample size for individuals over the age of 30 years was limited in white rhinos and nearly absent in black rhinos, thus restricting our interpretation of late-life DHEA patterns.

DHEA concentrations were higher in pregnant compared to non-pregnant females and increased progressively during gestation. With only a single year of monthly sampling, we were unable to capture an individual’s complete DHEA profile from conception to parturition, which limits the strength of conclusions that can be drawn regarding longitudinal hormone changes during pregnancy. However, our sample size was robust enough to determine that serum DHEA rises substantially throughout gestation and especially during mid-gestation to late gestation, with some white rhinos also showing a decrease in concentrations just prior to parturition. This pattern of consistently increasing DHEA concentrations contrasts with findings in mares, in which DHEA concentrations increase until mid-gestation before declining during late gestation (Legacki *et al.*, 2016; Satué *et al.*, 2019), and with human and non-human primates, in which DHEA rises only during late gestation (humans, Raeside, 2017; Japanese macaques, orangutans, howler monkeys, siamangs, Hart *et al.*, 2023). These differences possibly reflect species-specific sources of DHEA during pregnancy: in mares, the peak in maternal DHEA concentrations is attributed to the foetal gonads (Pashen and Allen, 1979; Pashen *et al.*, 1982; Legacki *et al.*, 2016, 2017; Conley and Ball, 2019), whereas in primates, the foetal adrenal gland is the likely source (Raeside, 2017; Hart *et al.*, 2023). The source of elevated maternal DHEA concentrations during rhino gestation remains uncertain, but the foetal gonads are a plausible contributor given the close evolutionary relationship between rhinos and horses.

Higher-frequency sampling across entire pregnancies is needed to confirm whether DHEA concentrations predictably drop before parturition in rhinos, as was observed for three individuals, and to better understand its biological source. If foetal in origin—via the foetal gonads, the foetal adrenals or both—DHEA may have value as an indicator of rhino foetal health. For example, Hart *et al.* (2023) found that faecal DHEA-S concentrations reliably distinguished successful from unsuccessful pregnancies in Japanese macaques, orangutans, siamangs and howler monkeys, although sensitivity varied depending on species and housing conditions. In the present study, the white rhino that experienced mid-gestation foetal loss did not have the gradual increase in DHEA concentrations observed in other pregnant individuals. Recognizing that this single observation should be interpreted with caution, it does echo findings in Japanese macaques, where faecal DHEA-S concentrations rise during late gestation in successful pregnancies but remain unchanged in failed ones (Takeshita *et al.*, 2016). Though preliminary, these patterns highlight a possible role for DHEA in assessing rhino foetal health.

The findings of this study provide further insight into the physiological roles of DHEA in black and white rhinos and contribute to a broader understanding of the species-specific differences common in rhino reproductive endocrinology. For instance, androgen and oestrogen metabolite concentrations accurately reflect estrous cycles in greater one-horned rhinos (Schwarzenberger *et al.*, 2000; Gómez *et al.*, 2004; Stoops *et al.*, 2004), but are unreliable indicators of cycling in other rhino species (Brown *et al.*, 2001; Roth *et al.*, 2018). Longitudinal monitoring of progesterone and its metabolites is therefore the standard for assessing reproductive cyclicity in black rhinos (Berkeley *et al.*, 1997; Garnier *et al.*, 2002), white rhinos (Schwarzenberger *et al.*, 1998; Patton *et al.*, 1999) and Sumatran rhinos (*Dicerorhinus sumatrensis*; Roth *et al.*, 2001, 2004), and for detecting pregnancy across all rhino species. Interestingly, faecal progestagen metabolite concentrations are higher in black than white rhinos during gestation (Schwarzenberger and Hermes, 2023), similar to serum DHEA concentrations in the present study.

Although DHEA concentrations increased consistently across rhino gestation, substantial individual-level variation and overlap between non-pregnant and early pregnant values limit the utility of single time-point measurements for pregnancy confirmation and highlight the importance of longitudinal sampling for interpreting DHEA patterns within individuals. For this reason, we did not attempt to derive an empirical pregnancy classification threshold from pregnant samples. Instead, a conservative reference concentration was defined using the upper 95th percentile of non-pregnant females and mapped onto the modelled gestational trajectory of DHEA concentrations to illustrate the timing of physiological divergence between non-pregnant and pregnant white rhinos.

Not all rhinos in managed care are trained for voluntary blood collections. Fortunately, DHEA can be evaluated using alternative matrices like faeces (e.g. Japanese macaques, Takeshita *et al.*, 2013) and urine (e.g. bonobos, *Pan paniscus*, Behringer *et al.*, 2012), and perhaps even less-studied matrices like saliva (e.g. humans, Gallagher *et al.*, 2006), hair (e.g. horses, Placci *et al.*, 2020) and horn (black and white rhinos, Rebecca Evey, personal communication). Such efforts may prove particularly useful for investigating wildlife reproductive function in a less invasive manner. For instance, urinary DHEA concentrations in zoo-housed giant pandas (*Ailuropoda melanoleuca*) increase significantly at the time of peak estrus and thus are used to detect optimal fertility windows (Wilson *et al.*, 2022). In contrast, sustained DHEA elevations may be linked to reproductive dysfunction in some species (rats, *Rattus norvegicus*, Mahmoud *et al.*, 2018; polar bears, *Ursus maritimus*, Brandhuber *et al.*, 2023), a phenomenon potentially mediated by chronic adrenal activity, high concentrations of glucocorticoids and DHEA and subsequent glucocorticoid-induced reproductive suppression (Dobson and Smith, 2000; Whitham *et al.*, 2020). Given the well-documented history of aberrant cyclicity in rhinos (Schwarzenberger *et al.*, 1998; Patton *et al.*, 1999), a better understanding of the relationship between DHEA and reproductive function may help identify causes of such aberrations and refine *ex situ* rhino breeding programmes.

In conclusion, this study provides the first validated method for measuring serum DHEA in rhinos and demonstrates that DHEA concentrations vary with species, age and pregnancy status. Although overlap between non-pregnant and early pregnant concentrations and substantial individual-level variation indicate that DHEA should not be interpreted in isolation, the observed pregnancy- and age-associated patterns provide new insight into the broader understanding of rhino endocrinology. Together, these findings support the potential utility of DHEA as a pregnancy monitoring tool in rhinos and establish a foundation for future studies integrating DHEA with additional physiological measures of animal health and well-being.

## Supplementary Material

Web_Material_coag007

## Data Availability

The data supporting this study’s findings are available from the corresponding author upon reasonable request. Any identifying information will be removed to protect the anonymity of participating facilities.
