# Supplementary material for: Pregnancy- and age-associated variation in serum dehydroepiandrosterone concentrations in black and white rhinoceroses
Source: Conserv Physiol. 2026 Feb 12;14(1):coag007. doi: 10.1093/conphys/coag007 (PMC12894765; doi:10.1093/conphys/coag007)
Supplement: Web_Material_coag007 [file web_material_coag007.zip › Supplementary_Figure1.pdf]

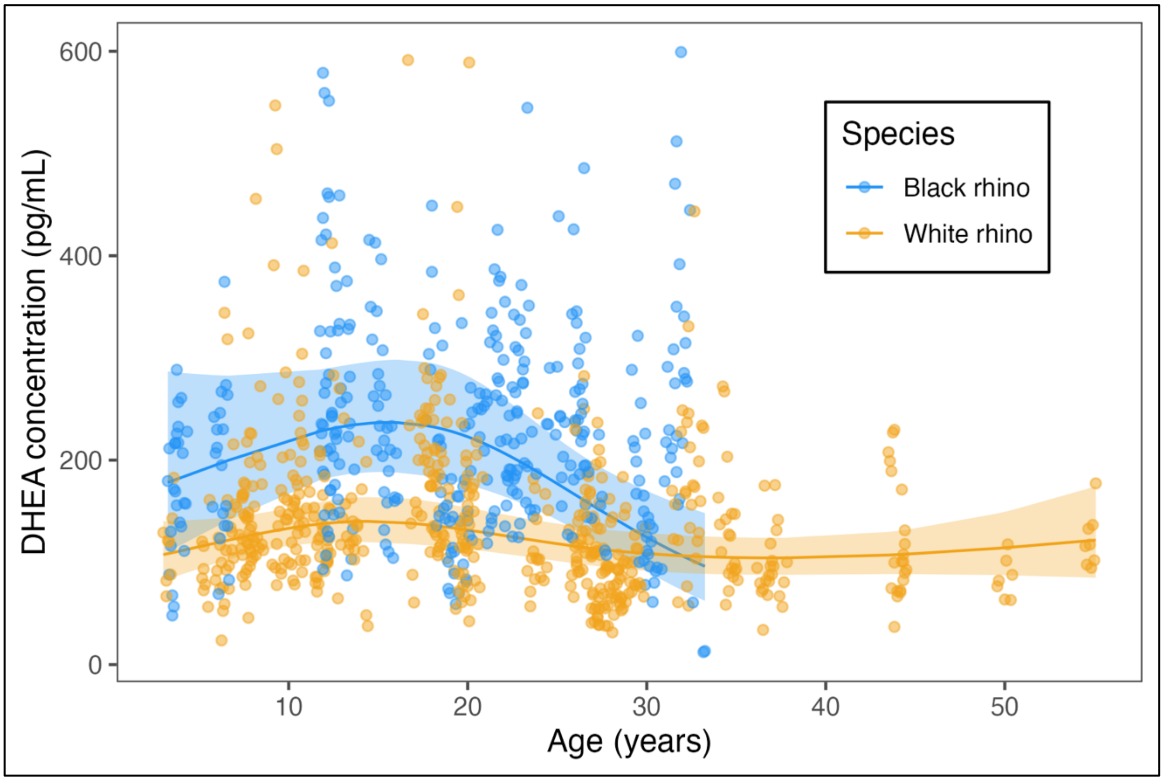

Supplementary Figure 1: Associations between age and serum DHEA concentrations in male and non-pregnant female rhinos. Lines indicate predicted DHEA concentrations based on monthly sampling, whereas circles indicate all monthly samples collected from 39 black and 67 white rhinos.
