# Supplementary material for: Pregnancy- and age-associated variation in serum dehydroepiandrosterone concentrations in black and white rhinoceroses
Source: Conserv Physiol. 2026 Feb 12;14(1):coag007. doi: 10.1093/conphys/coag007 (PMC12894765; doi:10.1093/conphys/coag007)
Supplement: Web_Material_coag007 [file web_material_coag007.zip › Supplementary_Table1.pdf]

Supplementary Table 1: Study population demographics and the number of serum samples collected for participating black rhinoceros (n = 22 male, 18 female) and white rhinoceros (n = 25 male, 46 female). Individuals with two Institutional IDs were transferred from the first facility to the second facility during the one-year collection period.

Rhino demographics					Number serum samples		
Institution ID	Rhino ID	Species	Age (years)	Sex	Total	Pregnant	Non-pregnant
1	23728998	White rhino	27	Male	8	-	-
1	14662651	White rhino	36	Male	11	-	-
1	22187249	White rhino	26	Female	11	0	11
1	22187234	White rhino	27	Female	12	0	12
1	22187246	White rhino	26	Female	10	0	10
2	19298676	Black rhino	18	Male	4	-	-
2, 19	14662333	Black rhino	20	Male	12	-	-
3	20563574	White rhino	9	Male	12	-	-
3	20563577	White rhino	36	Female	11	0	11
5	22939949	Black rhino	6	Male	8	-	-
6, 11	14662147	Black rhino	11	Male	9	-	-
6	19030328	Black rhino	5	Female	11	0	11
6	19030325	Black rhino	3	Female	12	0	12
6	14662354	Black rhino	11	Female	12	0	12
8	14662231	Black rhino	32	Male	3	-	-
8	19030340	Black rhino	11	Female	9	0	9
10	14662249	Black rhino	29	Male	11	-	-
10	14662507	White rhino	28	Male	8	-	-
10	14662510	White rhino	28	Female	8	5	3
10	14662513	White rhino	28	Female	12	0	12
10	14662540	White rhino	18	Female	9	0	9
10	21286957	White rhino	7	Female	9	0	9
14, 25	14662345	Black rhino	17	Male	11	-	-
14	14662351	Black rhino	12	Female	12	0	12
15	21385660	Black rhino	22	Male	12	-	-
15	21385657	Black rhino	29	Female	11	0	11
15	21385654	Black rhino	19	Female	11	0	11
16	14662255	Black rhino	29	Male	6	-	-
18	19990829	Black rhino	21	Male	11	-	-
18	19990805	Black rhino	20	Male	9	-	-
18	19990832	White rhino	26	Male	9	-	-

18	19990811	White rhino	23	Female	11	0	11
18	14662504	White rhino	26	Female	12	0	12
18	19990808	White rhino	12	Female	12	0	12
18	19990835	White rhino	7	Female	12	0	12
19	20285048	Black rhino	21	Male	11	-	-
19	14662534	White rhino	19	Male	8	-	-
19	24403797	White rhino	9	Male	12	-	-
19	20285051	Black rhino	31	Female	12	0	12
19	17244386	Black rhino	25	Female	11	0	11
19	17244422	Black rhino	23	Female	12	0	12
19	24403812	White rhino	23	Female	7	0	7
19	24403800	White rhino	11	Female	10	10	0
19	24403809	White rhino	5	Female	12	9	3
19	24403803	White rhino	43	Female	9	0	9
20	14662477	White rhino	25	Male	11	-	-
20	24421821	White rhino	17	Female	9	1	8
21	14662288	Black rhino	26	Male	9	-	-
21	19990814	Black rhino	26	Male	6	-	-
21	14662309	Black rhino	25	Female	7	0	7
22	14662654	White rhino	34	Male	12	-	-
22	14662501	White rhino	27	Female	12	0	12
22	14662624	White rhino	43	Female	6	0	6
24	19990823	Black rhino	31	Male	12	-	-
24	19990820	Black rhino	11	Male	12	-	-
24	14662342	Black rhino	18	Female	12	0	12
24	19990817	Black rhino	15	Female	12	3	9
25	24688568	Black rhino	3	Male	6	-	-
25	24422649	Black rhino	17	Female	3	0	3
26	19947173	White rhino	54	Male	8	-	-
26	19940654	White rhino	26	Female	11	0	11
26	19940438	White rhino	13	Female	11	0	11
26	19939259	White rhino	11	Female	11	11	0
27	14662276	Black rhino	27	Male	5	-	-
29	19030346	White rhino	6	Male	12	-	-
29	19030349	White rhino	6	Male	12	-	-
37	21850740	White rhino	17	Male	11	-	-
37	21850731	White rhino	17	Male	10	-	-
40	19030319	Black rhino	14	Female	9	0	9
41	24428154	Black rhino	19	Male	6	-	-

41	24428157	Black rhino	22	Female	6	0	6
42	15458923	White rhino	19	Male	5	-	-
42	15458941	White rhino	49	Female	7	0	7
43	19073289	White rhino	18	Male	9	-	-
43	17335124	White rhino	5	Female	6	0	6
45	14662498	White rhino	25	Male	4	-	-
45	19548899	White rhino	18	Female	5	3	2
47	22201313	White rhino	32	Male	6	-	-
47	22201325	White rhino	7	Female	8	5	3
47	22201316	White rhino	7	Female	6	0	6
49	24428346	Black rhino	14	Male	12	-	-
50	19024655	Black rhino	24	Female	9	0	9
51	24215274	Black rhino	3	Male	8	-	-
52	14662486	White rhino	32	Male	10	-	-
52	14662558	White rhino	12	Female	10	9	1
52	14662570	White rhino	12	Female	5	0	5
52	14662576	White rhino	10	Female	11	5	6
52	14662579	White rhino	9	Female	12	12	0
52	14662582	White rhino	8	Female	11	7	4
52	14662588	White rhino	8	Female	10	7	3
54	24212772	White rhino	16	Male	12	-	-
54	14662585	White rhino	9	Female	12	8	4
54	14662591	White rhino	9	Female	8	0	8
55	14662153	Black rhino	11	Male	5	-	-
55	14662492	White rhino	31	Male	11	-	-
55	14662156	Black rhino	11	Female	11	11	0
55	19030310	White rhino	10	Female	10	0	10
55	19030316	White rhino	11	Female	8	0	8
55	19030313	White rhino	11	Female	10	0	10
56	24429936	White rhino	34	Male	8	-	-
56	24429882	White rhino	11	Male	8	-	-
56	24429951	White rhino	7	Female	6	0	6
56	24429918	White rhino	7	Female	9	5	4
56	24429933	White rhino	7	Female	5	0	5
57	14662528	White rhino	19	Male	10	-	-
57	23577495	White rhino	43	Female	6	0	6
58	23217358	White rhino	19	Male	9	-	-
58	23217481	White rhino	19	Female	9	7	2
58	23217421	White rhino	19	Female	8	0	8

58	23217244	White rhino	19	Female	6	6	0
58	23215720	White rhino	3	Female	8	0	8
